# The Influence of the Solar Coronal Radiation on Coronal Plasma Structures, I: Determination of the Incident Coronal Radiation

**DOI:** 10.1007/s11207-018-1255-z

**Published:** 2018-02-09

**Authors:** Gerrard M. Brown, Nicolas Labrosse

**Affiliations:** SUPA, School of Physics and Astronomy, University of Glasgow, Glasgow, G12 8QQ UK

**Keywords:** Corona, quiet, Spectrum, ultraviolet

## Abstract

Coronal structures receive radiation not only from the solar disc, but also from the corona. This height-dependent incident radiation plays a crucial role in the excitation and the ionisation of the illuminated plasma. The aim of this article is to present a method for computing the detailed incident radiation coming from the solar corona, which is perceived at a point located at an arbitrary height. The coronal radiation is calculated by integrating the radiation received at a point in the corona over all of the corona visible from this point. The emission from the corona at all wavelengths of interest is computed using atomic data provided by CHIANTI. We obtain the spectrum illuminating points located at varying heights in the corona at wavelengths between 100 and 912 Å when photons can ionise H or He atoms and ions in their ground states. As expected, individual spectral lines will contribute most at the height within the corona where the local temperature is closest to their formation temperature. As there are many spectral lines produced by many ions, the coronal intensity cannot be assumed to vary in the same way at all wavelengths and so must be calculated for each separate height that is to be considered. This code can be used to compute the spectrum from the corona illuminating a point at any given height above the solar surface. This brings a necessary improvement to models where an accurate determination of the excitation and ionisation states of coronal plasma structures is crucial.

## Introduction

Structures located in the Sun’s corona, such as prominences, loops, streamers, or spicules, receive light directly from the solar disc and from the surrounding coronal plasma. In the case of prominences, for instance, it has been well known since the work of Hirayama (1963) that the incident radiation is crucial for determining the excitation and ionisation of the prominence plasma, particularly at wavelengths below the H ionisation threshold ($\lambda < 912$ Å). Its importance has been further discussed by, *e.g.*, Labrosse *et al.* (2010), Heinzel (2015), and Labrosse (2015).

In order to assess the influence of the radiation coming from the solar corona on the radiative processes within coronal structures, it is necessary to determine the characteristics of the incident radiation from the corona incident on a point at an arbitrary height above the solar surface. While it is possible to estimate the coronal spectrum illuminating a region of interest based on an appropriate set of observations (see, *e.g.*, Andretta *et al.* 2008, where the EUV coronal back-radiation on an active region was estimated), it is not practical to do so for an arbitrary height and under arbitrary conditions. In the general case, the coronal spectrum incident on a structure located in the solar corona must therefore be computed using our best knowledge of atomic data. As far as we are aware, this has not been done for an arbitrary height inside the corona before.

In this article, we present a method for computing the coronal spectrum illuminating a point located in the quiet solar corona at an arbitrary height, based on atomic data provided by CHIANTI (v7: Dere *et al.* 1997; Landi *et al.* 2012). In Section 2 we detail the set-up adopted for the calculations. In Section 3 we explain how our code was tested. Finally, we present our results in Section 4 and our conclusions in Section 5.

## Method

### Equations

The intensity of a spectral line at wavelength $\lambda $ along one path through a medium can be given as

1 $$ I(\lambda ) = \int_{0}^{l} \frac{G(T) Ab\, n_{\mathrm{e}}^{2}}{4 \pi } \,\mathrm{d}r , $$

where $l$ is the total length of the path, $r$ is the distance along the path, $Ab$ is the abundance of the element relative to hydrogen, and $n_{\mathrm{e}}$ is the electron number density. We take the contribution function to be

2 $$ G(T) = \frac{h c}{\lambda } \frac{A_{ji}}{n_{\mathrm{e}}} \frac{n _{j}}{N_{\mathrm{ion}}} \frac{N_{\mathrm{ion}}}{N_{ \mathrm{el}}} \frac{N_{\mathrm{H}}}{n_{\mathrm{e}}} , $$

where $A_{ji}$ is the spontaneous emission coefficient from level $j$ to level $i$, $n_{j}$ is the number density for excited level $j$, $N_{\mathrm{ion}}$ is the number density of the ion, $N_{ \mathrm{el}}$ is the number density of the element, and $N_{\mathrm{H}}$ is the number density of hydrogen.

**Figure 1:**
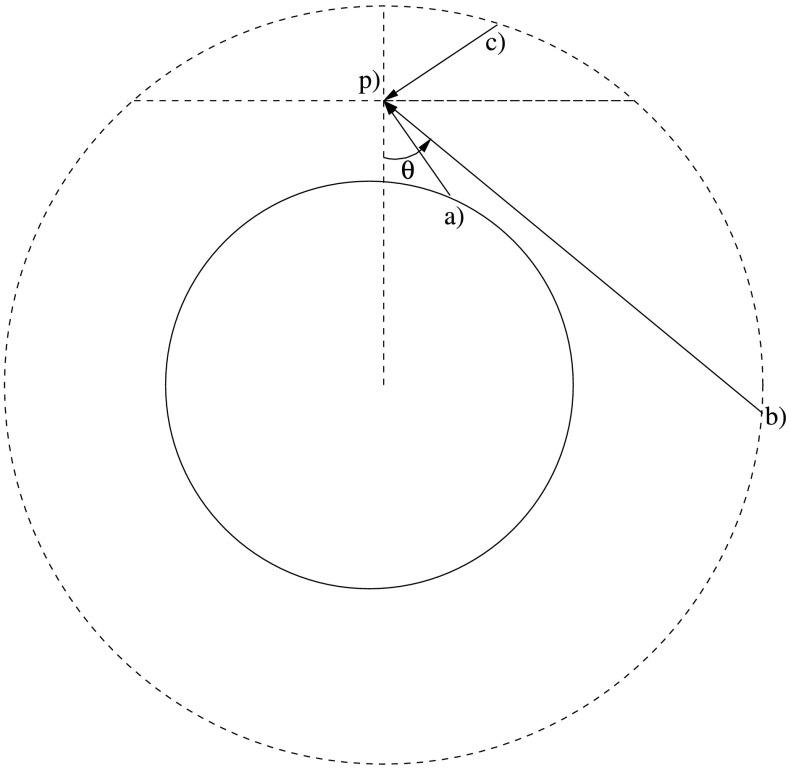
Three types of paths through the corona from various starting positions to point P, which receives the coronal radiation. These three types have to be considered. Path a increases in height from the initial point on the solar surface until it terminates on point P, path b decreases in height from the initial point until it reaches the point of closest approach where the path is parallel to the solar surface, after which it increases in height until it terminates at point P, and path c decreases in height from the highest considered point in the corona until it terminates at point P. $\theta $ is the angle between the normal to the solar surface and the path.

Of course, the prominence is illuminated from all directions in the corona, and this radiation will be different for different directions. The length of the path will be different for different directions throughout the corona, as seen in Figure 1. Moreover, the intensity depends on temperature and density values along each path.

**Figure 2:**
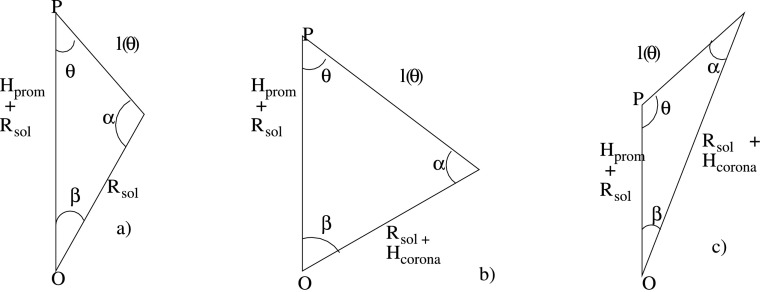
Trigonometric determination of the length of path $l(\theta )$ for the three cases shown in Figure 1 from the height of the prominence $H_{\mathrm{prom}}$, the radius of the Sun $\mathrm{R}_{\odot }$, the maximum height of the corona considered $H_{\mathrm{corona}}$, and the angle between the path and the normal to the solar surface. Point P is the point illuminated by the corona being considered, point O is the centre of the Sun. The angles $\alpha $ and $\beta $ can be determined from the known angles and lengths to assist in determining $l(\theta )$.

The path length that must be considered is the length [$l( \theta )$], which can be determined using sine and cosine rules by considering the triangles in Figure 2. The mean intensity coming from all directions is then

3 $$ J(\lambda ) = \frac{1}{4 \pi } \int_{0}^{2\pi } \int_{0}^{\pi } I( \lambda ) \sin(\theta ) \, \mathrm{d}\theta \, \mathrm{d}\phi . $$

Therefore, the mean intensity received at a given point in the corona is

4 $$ J(\lambda ) = \frac{1}{16 \pi^{2} } \int_{0}^{2\pi } \int_{0}^{\pi } \int_{0}^{l(\theta )} Ab\, G(T) n_{\mathrm{e}}^{2} \, \mathrm{d}r \sin(\theta ) \, \mathrm{d}\theta \, \mathrm{d}\phi \ . $$

The mean intensity from all lines in the wavelength range of interest for a given continuum can be combined to compute the photoionisation rate coefficient [$R$] due to coronal radiation using

5 $$ R = 4 \pi \int_{\nu_{0}}^{\infty } \frac{ \alpha_{\nu } J(\nu ) }{ h \nu }\, \mathrm{d}\nu , $$

where $\nu_{0}$ is the threshold frequency for ionisation, $\alpha ( \nu )$ is the photoionisation cross-section, and $J(\nu )$ is the mean intensity in the line.

### Application

The contribution function [$G(T)$] contains the atomic information necessary to obtain that line’s intensity. It is obtained for each of the lines in the wavelength range being considered from the CHIANTI atomic database.

The CHIANTI atomic database (Dere *et al.* 1997; Landi *et al.* 2012) can provide the intensity of spectral lines over a desired wavelength range from a set of input parameters (usually temperature and density). However, because we are interested in calculating the intensity falling on a point located within the solar corona itself rather than the intensity as observed from a point external to the corona, we use the CHIANTI ch_synthetic.pro routine to calculate the contribution function. This then yields the mean intensities of interest according to Equation 4.

**Figure 3:**
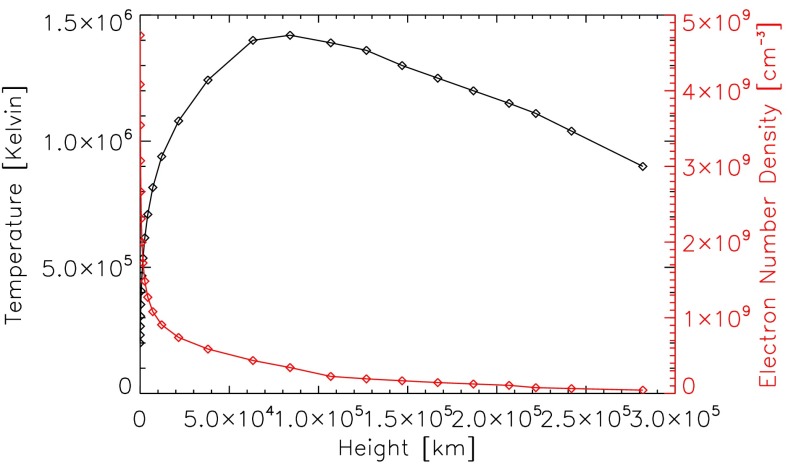
Variation in temperature and electron density through the corona based on the quiet-Sun corona model of Fontenla *et al.* (2011).

**Figure 4:**
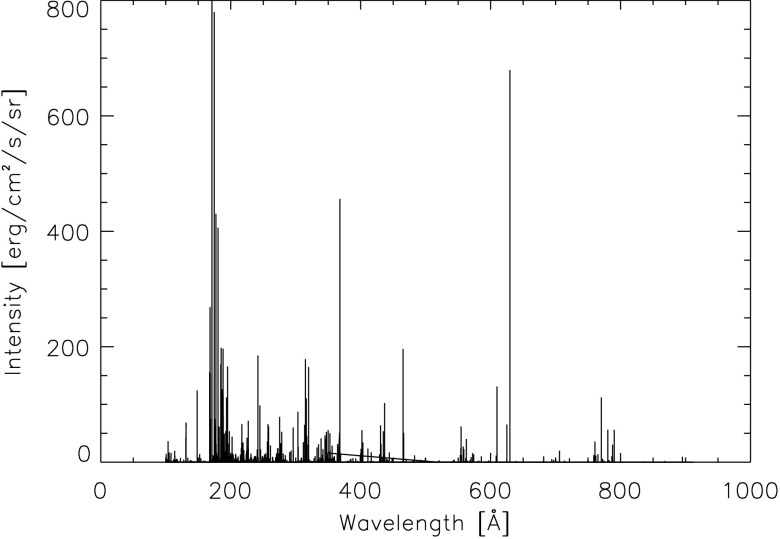
The total mean intensity of 25,020 coronal lines between 100 and 912 Å received at a height of 10,000 km in the corona.

A temperature and density profile of the corona is obtained from Fontenla *et al.* (2011). The contribution function depends on the density and temperature, so that the contribution function for each density and temperature needs to be obtained before we can use it in Equation 4 for each line to be calculated. Fontenla *et al.* (2011) provide profiles of the temperature and density through the corona for different solar conditions. The model that we are using in this study is that of the quiet-Sun inter-network for the corona, referred to by Fontenla *et al.* (2011) by the reference model number 1011, which contains values from 2000 km above the solar surface to 282,000 km above the solar surface. This represents the conditions of the majority of the solar corona for periods of low solar activity. The temperature and density variations of this profile with height can be seen in Figure 3. The coronal lines between 100 and 912 Å resulting from this model, illuminating a point in the corona at a height of 10,000 km, can be seen in Figure 4. A total of 25,020 line transitions were considered between 100 and 912 Å. Figure 4 shows that we can now obtain detailed information on the mean intensities of lines emitted by the solar corona as viewed from a point located at an arbitrary height in the corona.

Our code considers the corona as being split into two regions: one region consists of all of the type-a paths in Figure 1, and the other region consists of all other paths. This is to take into account the discontinuity that occurs when we transition from type-a paths to type-b paths. In the calculations of the mean intensity presented in this article, 500 paths within each region were considered for a total of 1000 paths through the corona. A number of equally spaced points were placed along each path. The temperature and density at each point were interpolated from the coronal profile. In the calculations of the mean intensity presented in this article, 1000 points along each path were used. We took the atmosphere to be spherically symmetric with respect to the solar centre, so that the integration in $\phi $ can be dealt with through a multiplication by $2\pi $.

## Verification

Our calculations require verification that they provide the correct values for the mean intensities of the spectral lines illuminating a point in the solar corona. For this, we first set up our code to calculate line intensities emerging from the corona as seen by an observer from outside, and compare them with intensities obtained from CHIANTI v7 (Landi *et al.* 2012).

We have used the CHIANTI ch_synthetic.pro procedure to calculate the contribution functions. CHIANTI can also compute intensities when provided with the differential emission measure (DEM), ionisation fractions, and the densities and temperatures in the model atmosphere:

6 $$ \mathrm{DEM} = n_{\mathrm{e}}^{2} \frac{\mathrm{d} r}{\mathrm{d} T} . $$

A DEM can be used to obtain an intensity through

7 $$ I(\lambda ) = \int_{T_{A}}^{T_{B}} \frac{G(T) Ab \, \mathrm{DEM}}{4 \pi } \,\mathrm{d}T , $$

which is integrated from the lowest temperature value of the model atmosphere [$T_{A}$] to the highest value of the model atmosphere [$T_{B}$].

This intensity is for a single path through the atmosphere, while our calculations are over all directions (Equation 4). To compare our calculations to CHIANTI, an integration over only one path as in Equation 1 was performed. Equation 6 can be rearranged to

8 $$ \mathrm{DEM}\, \mathrm{d}T = n_{\mathrm{e}}^{2}\, \mathrm{d}r , $$

with the other terms present in the integration [${G(T) Ab}/{4 \pi }$], this shows the integrations in Equations 1 and 7 to be equivalent:

9 $$ \frac{G(T) Ab\, \mathrm{DEM}}{4 \pi }\, \mathrm{d}T = \frac{G(T) Ab\, n_{\mathrm{e}}^{2}}{4 \pi } \,\mathrm{d}r . $$

Line intensities emitted by the Fontenla *et al.* (2011) quiet-Sun corona are compared between our calculations and CHIANTI over a range of wavelengths from 550 to 600 Å along one path straight down the corona from a height in the coronal model at which the sign of the temperature gradient changes (63,000 km) down to the lowest height in the coronal model (2,000 km). Only a smaller range of wavelengths from 550 to 600 Å was considered instead of the full range from 100 to 912 Å, as we deemed it more practical to compare over a smaller range rather than the full range. To ensure that CHIANTI considers the same atmosphere as we do in our calculations, we needed to provide a new DEM calculated for the CHIANTI software, with Equation 6 from the same values of height, temperature, and density as we used in our calculations. CHIANTI also required a list of temperatures and densities to use in place of a constant density.

**Figure 5:**
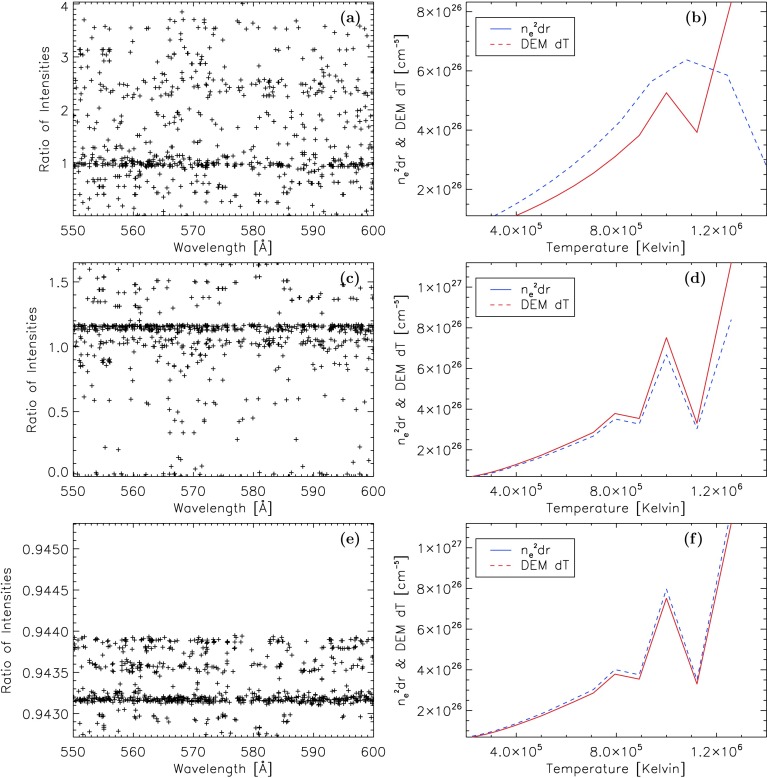
**a** Ratio between the intensities derived from CHIANTI and those calculated by our code over all lines for the case of the Fontenla *et al.* (2011) quiet-Sun atmosphere. **b** Comparison between $n_{\mathrm{e}}^{2}\, \mathrm{d}r$ and $\mathrm{DEM} \,\mathrm{d}T$ for where our code used the Fontenla *et al.* (2011) Quiet-Sun atmosphere. **c** and **d** Same as **a** and **b**, but with modified data points. **e** and **f** Same as **c** and **d**, but using rectangular numerical integration.

Figure 5a shows the ratios between the line intensities produced by our calculation and those by CHIANTI. Our investigation of the discrepancy between the two methods revealed that all spectral lines from the same ion of the same element have the same ratio of integrated intensities between the two methods. Mathematically, the methods used in both calculations are equivalent, as shown by Equations 8 and 9. The two sides of Equation 8 are plotted in Figure 5b with $n_{\mathrm{e}}^{2} \,\mathrm{d}r$ values coming from the values of $n_{\mathrm{e}}^{2}$ and $\mathrm{d}r$ used in our calculations, and $\mathrm{DEM}\,\mathrm{d}T$ values coming from the values of $\mathrm{DEM}$ and $\mathrm{d} T$ used in CHIANTI, based on the same quiet-Sun corona model.

The discrepancy visible in Figure 5b shows that the disagreement comes from the numerical values used in the calculations. The ch_synthetic.pro routine uses data points whose logarithmic temperatures are at 0.05 intervals. If the DEM it is given does not have the same temperature intervals, then it interpolates the DEM at these 0.05 intervals within the range of temperatures in the DEM. The data points in the coronal profile that we used in both our calculations and in creating the DEM that we supplied CHIANTI with do not have the same intervals in temperature. In order for Equation 8 to hold true, the temperature intervals must be the same. Therefore, a new coronal profile for our calculations was created, using the same temperature intervals as CHIANTI, with the values at these points interpolated from the original profile.

There is still a discrepancy after this change. Figure 5c shows that there is still a noticeable spread in the ratio of the line intensities. Figure 5d shows the cause of this. Although the $n_{\mathrm{e}}^{2} \,\mathrm{d}r$ and $\mathrm{DEM} \, \mathrm{d}T$ curves are now the same shape, they still slightly differ in value.

This discrepancy is found to be in the way the two methods handle the numerical integration of the intensity. The CHIANTI ch_synthetic.pro routine uses the rectangle method of numerical integration, whilst our calculations have used the trapezoidal rule. The rectangle method approximates the area below the curve as a series of rectangles with their bases on the $x$-axis and one of their top corners on the curve. The trapezoidal rule uses trapezoids that differ from the rectangles in that both of the upper corners lie on the curve. The use of two different approximations of the area below a curve gives two different values for the integration. Our calculations were changed for the final comparison to use the rectangle method of numerical integration. The line intensities of our calculations are now much closer to the line intensities of CHIANTI. Figure 5e shows the ratios of the line intensities, which show much better agreement than the previous comparisons. The agreement is now close enough that there can be confidence in the calculations.

In the rest of this work we used the trapezoidal rule of numerical integration. The Fontenla *et al.* (2011) quiet-Sun coronal profile was used without the modified intervals in the rest of this work.

## Illumination by Coronal Lines on a Point in the Solar Atmosphere

In the previous section, we extensively discussed the comparisons that we have made with CHIANTI to demonstrate that when all possible sources of discrepancies were removed, our code and CHIANTI yield identical results when the radiation from the quiet-Sun corona is computed as if it were observed from outside the corona (Figure 5). In this study, however, our main goal is to model the optically thin radiation from the outer corona that illuminates a point located at any given height in the solar corona. To do this, we use the relevant geometrical set-up described in Section 2 to compute the coronal radiation as seen by a point located within the solar corona at an arbitrary height above the surface. We present our results for various strong lines at wavelengths lying in the H and He resonance continua. These strong lines are in principle able to significantly affect the excitation and ionisation state of the plasma.

### Variation of the Incident Spectrum with Height

As can be understood from looking at Figure 1, the spectrum needs to be calculated for the different heights of interest. Points at different altitudes will be illuminated by different portions of the solar corona. There will also be a stronger influence from regions of the corona closer to the vantage point.

**Figure 6:**
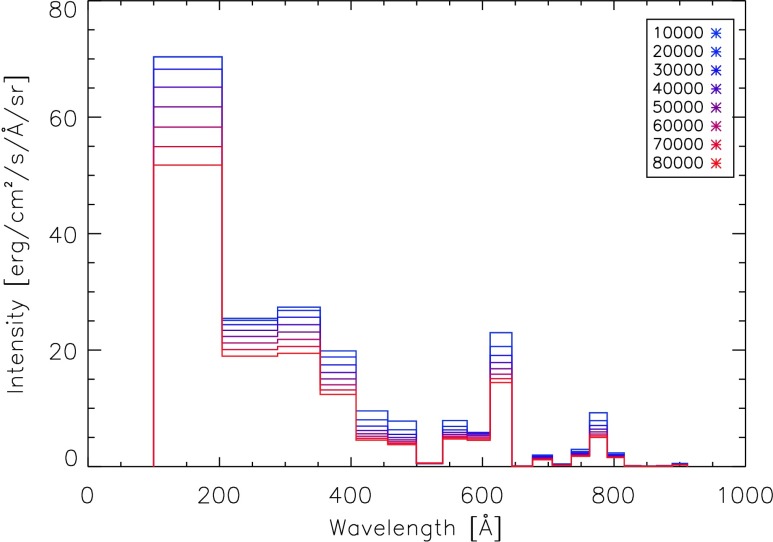
Spectrum summed into bins for eight different heights in the corona [km].

These two effects can be seen in Figure 6, which shows the binned spectrum as seen at eight different heights from 10,000 to 80,000 km. The size of the bins decreases with increasing wavelength to reflect the frequency dependence of the hydrogen photoionisation cross-section. The intensity in each bin varies in a different way for each bin. Different bins are dominated by lines from different ions. If a bin is dominated by lines of a given ion, the intensity of the bin will be at its greatest at the height at which the line intensities for that ion peak. The numerical values of the bins shown in Figure 6 can be found in Table 1.

**Table 1:** Total intensity from coronal lines illuminating a point at various heights within the corona.

Wavelength range [Å]	Intensity [erg cm^−2^ s^−1^ sr^−1^] at a height [km]							
	10,000	20,000	30,000	40,000	50,000	60,000	70,000	80,000
100 – 203	7307	7310	7088	6767	6417	6055	5707	5377
203 – 288	2148	2119	2056	1975	1887	1793	1697	1602
288 – 353	1773	1737	1661	1578	1496	1415	1326	1260
353 – 407	1085	1028	953	882	822	767	719	673
407 – 455	460	386	335	298	271	250	233	218
455 – 499	340	274	240	217	199	185	174	163
499 – 539	20	20	21	22	24	24	24	22
539 – 576	294	256	234	218	205	194	185	176
576 – 611	189	204	204	197	187	177	167	158
611 – 644	761	682	631	591	555	524	499	476
644 – 676	2	3	2	2	2	2	2	2
676 – 706	59	56	51	47	43	40	37	35
706 – 735	13	11	9	8	8	7	7	6
735 – 762	82	71	65	60	57	53	50	48
762 – 789	247	211	189	172	160	149	141	133
789 – 815	61	54	50	47	45	42	41	40
815 – 840	4	3	3	3	3	3	2	3
840 – 865	2	2	1	1	1	1	1	1
865 – 888	4	3	3	3	2	2	2	2
888 – 911	12	10	9	8	7	7	6	6

Some individual lines show how changing the height affects their mean intensities. This is connected to the variation of the temperature with height in the corona (see Table 2).

**Table 2:** Temperature at several heights in the corona.

Height [km]	log*T* [K]
10,000	5.948
20,000	6.024
30,000	6.066
40,000	6.099
50,000	6.120
60,000	6.140
70,000	6.148
80,000	6.151

**Figure 7:**
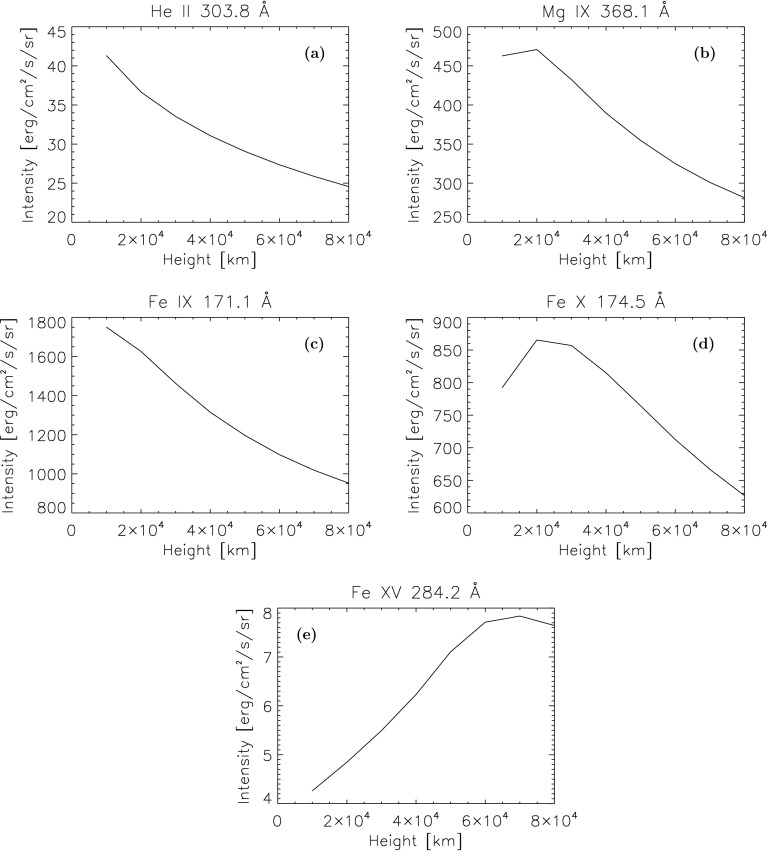
Variation in total intensity with height for the He ii 304 Å, Mg ix 368 Å, Fe ix 171 Å, Fe x Å, and Fe xv Å lines.

The He ii 304 line in Figure 7a is formed at a lower temperature ($\log T=4.95$) than any of the coronal temperatures considered. Hence its mean intensity is greatest for the lowest height in the corona (*i.e.* at the lowest local temperature).

The variation of the Mg ix 368 Å line mean intensity with height is shown in Figure 7b. The formation temperature given by CHIANTI is $\log T = 6$, which is closest to the temperature of the corona at the height of 20,000 km (Table 2 and Figure 3). At greater heights, the total mean intensity of the line decreases due to the increase in temperature.

Figure 7c shows the variation in mean intensity against height for the Fe ix 171 line ($\log T = 5.95$). As expected, the mean intensity is strongest at 10,000 km and decreases with height. The Fe x 174 Å line has a formation temperature of $\log T = 6.05$, which would correspond to an altitude between 20,000 and 30,000 km. The mean intensity of this line, shown in Figure 7d, is at its greatest at these heights. Continuing this trend of lines with a higher formation temperature having their greatest value at higher heights is the Fe xv 284 Å line ($\log T = 6.35$), shown in Figure 7e. Its formation temperature is greater than the temperature at any of the heights considered.

Although the mean intensities of the lines are integrated over the entire corona, the portion of the corona nearest to the point considered has the greatest effect on the incident spectrum. The mean intensity of each of these lines is strongest when viewed from a point in the corona closest to where they are formed.

**Figure 8:**
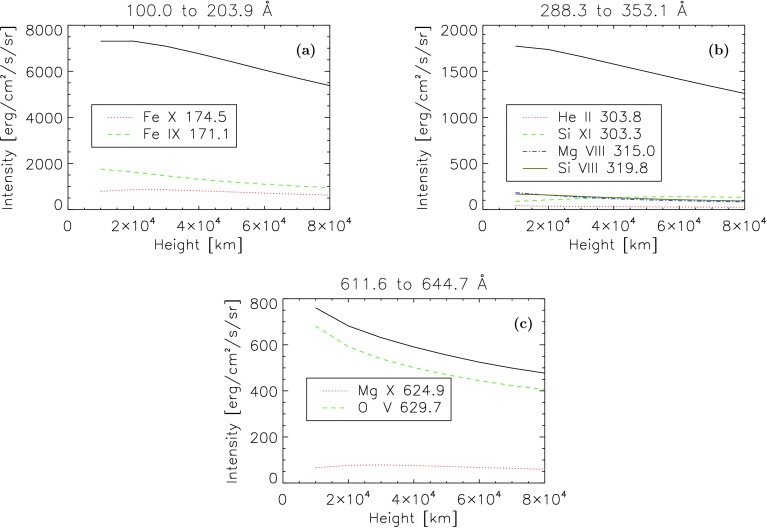
Variation in total mean intensity of all lines in a given height range for the range 100 – 203 Å **a**, 289 – 254 Å **b**, and 612 – 644 Å **c**. The variation of some individual lines with intensities greater than 10% of the interval they are in is also plotted for comparison.

Figure 8 shows that the total mean intensity over a given wavelength range is more heavily influenced by some lines rather than others. These plots show the total mean intensity for all lines within the wavelength range, and the total mean intensity for lines within this range that each make up more than 10% of the total mean intensity of all lines within the range. Each wavelength range features about two to five of these lines. Figure 8a shows a wavelength interval (100 – 203 Å) that is mostly made of lines weaker than the 10% of the total intensity of the interval required to be shown in the figure. Only two lines exceed 10% of the total mean intensity, so most of the light within this range will come from many lines rather than only a few of the strongest lines. Figure 8b shows a wavelength range (289 – 353 Å) that is shaped by more than just one line. Most of the four lines that are strongest in this range decrease with height, and the total mean intensity in this range also decreases with height. Figure 8c shows a wavelength range (612 – 644 Å) that is dominated by one line in particular. In this plot it is clear that the O v 630 Å line contributes most of the total mean intensity within this range.

Each line’s mean intensity will vary with height in a different way from other lines, which means that for different heights, each line will have to be recalculated. It is not possible to simply calculate each line once for one height and then multiply the whole spectrum by some scaling factor, as each line scales differently with height. Summing the lines across wavelength ranges would not solve this issue, as the total mean intensity of different wavelength ranges will also scale differently with height.

## Conclusions

This article presents a method for obtaining the radiation from the quiet-Sun corona as observed from a location within the corona. We showed that the radiation from the corona illuminating a point within the corona depends on the height of said point above the solar surface, and why it is necessary to calculate this coronal spectrum separately for different heights within the corona.

Section 2 detailed our calculations of the spectrum of the corona at different heights within the corona were performed. Contribution functions [$G(T)$] were obtained for electron densities at various heights through the corona. These contribution functions were obtained from the CHIANTI atomic database for all lines between 100 and 912 Å, and the coronal densities and temperatures used here are the temperatures and densities from the quiet-Sun model corona of Fontenla *et al.* (2011, model 1011). These contribution functions, densities, and temperatures were used in the integration of Equation 4 for each spectral line. The spectrum as seen from a height of 10,000 km in the corona that results from this calculation is shown in Figure 4

Our code was tested in Section 3 under some simple scenarios that enable direct comparisons with the CHIANTI spectral synthesis routines. Intensities for spectral lines along one path through the corona were calculated and compared to intensities provided by CHIANTI. Comparisons between the two methods revealed discrepancies that can be ascribed to differences in the computational methods.

In Section 4 we presented our results on the determination of illumination by coronal lines on a point in the solar atmosphere. The mean intensities of individual lines vary such that they are greatest when being observed from a height in the corona that is at a temperature closest to the formation temperature of the line. The fact that different lines have different formation temperatures means that the lines will not vary in height in the same way. This carries on to when lines are summed within wavelength bins. When considering the incident coronal radiation on a coronal structure at a certain height, it is necessary to calculate this coronal radiation separately for each height to be considered.

**Figure 9:**
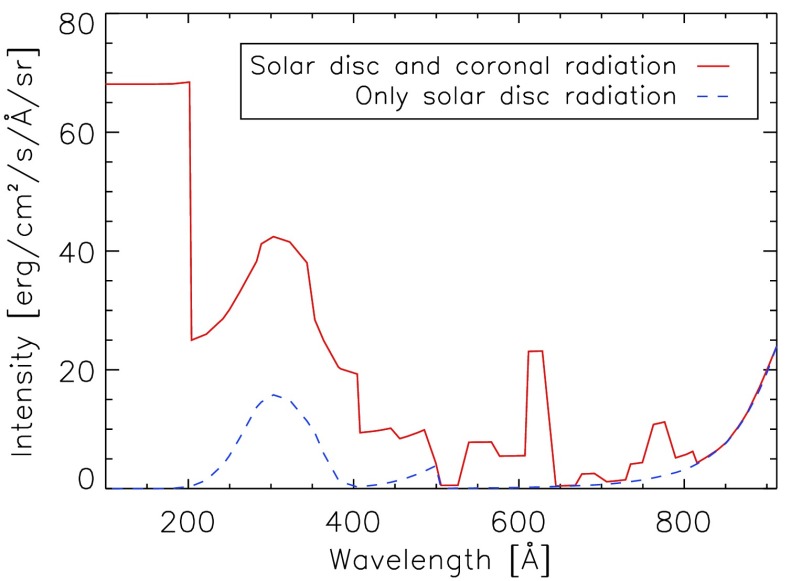
Comparison of the incident radiation used in the context of prominence modelling with the coronal radiation and without the corona radiation below 912 Å at a height of 10,000 km.

Figure 9 shows the difference that adding this coronal radiation makes to the incident spectrum used in the context of prominence modelling. In a forthcoming article, we will apply this method to the specific case of solar prominences, and investigate the effects of adding this radiation to prominence modelling, with particular attention to the ionisation of hydrogen and helium.
